# Soluble VCAM-1 promotes gemcitabine resistance via macrophage infiltration and predicts therapeutic response in pancreatic cancer

**DOI:** 10.1038/s41598-020-78320-3

**Published:** 2020-12-03

**Authors:** Ryota Takahashi, Hideaki Ijichi, Makoto Sano, Koji Miyabayashi, Dai Mohri, Jinsuk Kim, Gen Kimura, Takuma Nakatsuka, Hiroaki Fujiwara, Keisuke Yamamoto, Yotaro Kudo, Yasuo Tanaka, Keisuke Tateishi, Yousuke Nakai, Yasuyuki Morishita, Katsura Soma, Norihiko Takeda, Harold L. Moses, Hiroyuki Isayama, Kazuhiko Koike

**Affiliations:** 1 Department of Gastroenterology, Graduate School of Medicine, The University of Tokyo, Bunkyo-ku, Tokyo, 113-8655 Japan; 2 Division of Medical Research Planning and Development, Nihon University School of Medicine, Itabashi-ku, Tokyo, 173-8610 Japan; 3 Department of Molecular Pathology, Graduate School of Medicine, The University of Tokyo, Bunkyo-ku, Tokyo, 113-8655 Japan; 4 Department of Cardiovascular Medicine, Graduate School of Medicine, The University of Tokyo, Bunkyo-ku, Tokyo, 113-8655 Japan; 5 Division of Cardiovascular Medicine, Department of Medicine, Jichi Medical University School of Medicine, Tochigi, 329-0498 Japan; 6 Vanderbilt-Ingram Comprehensive Cancer Center, Vanderbilt University, 691 Preston Building, Nashville, TN 37232 USA; 7 Department of Gastroenterology, Juntendo University School of Medicine, 3-1-3 Hongo, Bunkyo-ku, Tokyo, 113-8431 Japan

**Keywords:** Pancreatic cancer, Cancer epidemiology, Cancer microenvironment, Cancer therapeutic resistance, Chemotherapy, Targeted therapies

## Abstract

Pancreatic cancer is one of the malignant diseases with the worst prognosis. Resistance to chemotherapy is a major difficulty in treating the disease. We analyzed plasma samples from a genetically engineered mouse model of pancreatic cancer and found soluble vascular cell adhesion molecule-1 (sVCAM-1) increases in response to gemcitabine treatment. VCAM-1 was expressed and secreted by murine and human pancreatic cancer cells. Subcutaneous allograft tumors with overexpression or knock-down of VCAM-1, as well as VCAM-1-blocking treatment in the spontaneous mouse model of pancreatic cancer, revealed that sVCAM-1 promotes tumor growth and resistance to gemcitabine treatment in vivo but not in vitro. By analyzing allograft tumors and co-culture experiments, we found macrophages were attracted by sVCAM-1 to the tumor microenvironment and facilitated resistance to gemcitabine in tumor cells. In a clinical setting, we found that the change of sVCAM-1 in the plasma of patients with advanced pancreatic cancer was an independent prognostic factor for gemcitabine treatment. Collectively, gemcitabine treatment increases the release of sVCAM-1 from pancreatic cancer cells, which attracts macrophages into the tumor, thereby promoting the resistance to gemcitabine treatment. sVCAM-1 may be a potent clinical biomarker and a potential target for the therapy in pancreatic cancer.

## Introduction

Pancreatic cancer is one of the most lethal malignant diseases with 6 months of median survival. It is the fourth leading cause of cancer-related deaths in Japan^1^ and will become the second in the U.S.A. by 2030^2^. The overall 5-year survival rate of the patients is 9%^3^. Most pancreatic cancer patients have an advanced disease at the time of diagnosis, in which case the efficacy of treatment, including chemotherapy, is limited^4^. One of the reasons pancreatic cancer patients have poor prognosis is an early acquisition of resistance to the chemotherapy. Thus, it is critical to know immediately when the disease is getting resistant to the current therapy so that the ineffective therapy is switched. However, there is no reliable biological markers to indicate the resistance to the therapy before we observe an increase in tumor burden by imaging. Although CA19-9 has been used as a marker for estimating tumor burden in pancreatic cancer patients, the level of CA19-9 in blood is often affected by nonspecific conditions^5^. Imaging can also be inaccurate because pancreatic cancer tissue is generally accompanied by abundant desmoplastic change and it is often difficult to distinguish tumor tissues and non-tumor tissues.

Based on pancreatic epithelium-specific *Kras* oncogene activation, several genetically engineered mouse models (GEMMs) for pancreatic cancer have been reported^6–10^, including the model we have previously reported^6^, which harbors Kras^G12D^ mutation and deletion of TGFβ receptor type II and can faithfully reproduce human pancreatic intraepithelial neoplasm (PanIN) and pancreatic ductal adenocarcinoma (PDAC) lesions. GEMMs have been broadly used for basic and translational research in this field because of their clinical relevance. These mouse models display abundant desmoplastic changes in the pancreas, as seen in the microenvironment of human pancreatic cancer, thus are considered suitable for researches in the preclinical settings^11,12^.

While vascular cell adhesion molecule-1 (VCAM-1), a cell adhesion molecule, is known to be expressed on endothelial cells in an inflammatory condition and helps adhesion of immune cells^13,14^, it has been suggested to have an important role in cancer biology. While VCAM-1 expressed on endothelial cells facilitates lung metastasis of ovarian carcinoma^15^, studies suggested VCAM-1 is expressed on cancer cells such as breast, renal, and gastric cancer^16–20^. Soluble form of VCAM-1 (sVCAM-1), which is cleaved from cell surfaces, is detected in the serum of several types of cancer patients and was reported as a staging or prognosis marker in several types of cancer^21–29^. VCAM-1 was found aberrantly overexpressed in resected PDAC tissues^30,31^. Expression of VCAM-1 in PDAC, evaluated with immunohistochemistry, was reported to be associated with poor prognosis^31^. However, the function and role of VCAM-1 and sVCAM-1 in advanced PDAC, which is the majority in PDAC patients, remains elusive.

In this study, we sought to find a reliable biomarker for resistance to chemotherapy, which can be measured repeatedly by blood test, using a genetically engineered mouse model and human plasma samples collected from pancreatic cancer patients with advanced diseases. We show sVCAM-1 induces resistance to gemcitabine treatment by attracting macrophages to tumor microenvironment, and sVCAM-1 in the plasma can be a reliable prognostic biomarker of chemotherapy for PDAC patients.

## Results

### Soluble VCAM-1 is released by pancreatic cancer cells in response to gemcitabine treatment

**Figure 1:**
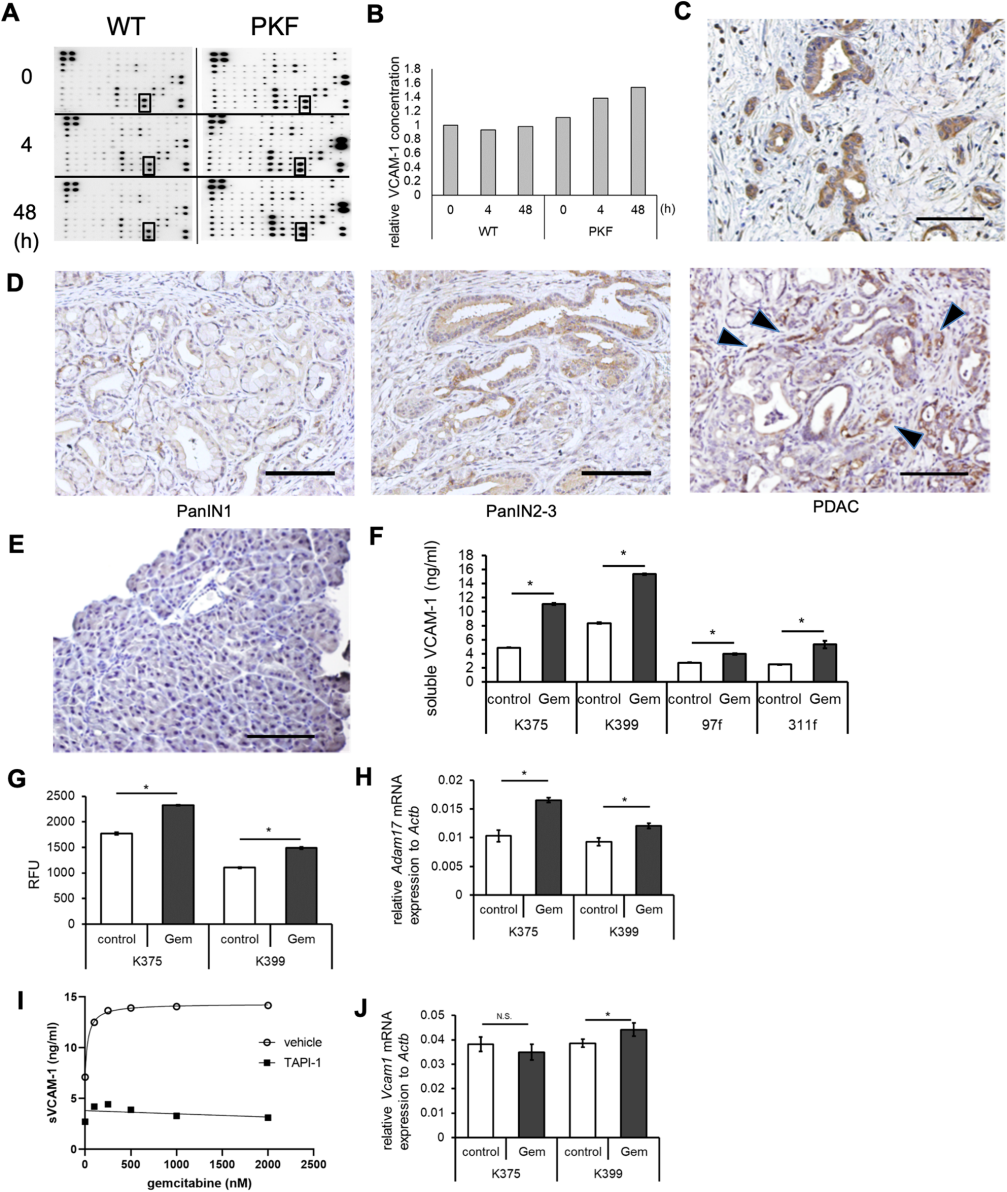
Pancreatic cancer cells release soluble VCAM-1 during gemcitabine treatment. (**A**) Whole pictures of membrane cytokine array 0 h, 4 h and 48 h after gemcitabine treatment. VCAM-1 spots are indicated in rectangles. WT: wild type. (**B**) Bar graph showing quantification of the density of VCAM-1 spots obtained from Fig. 1A. (**C**) Representative picture of immunohistochemical staining (IHC) for VCAM-1 in human pancreatic cancer. Scale bar, 100 µm. (**D**) Representative pictures of IHC for VCAM-1 in PanIN1, PanIN2-3, and PDAC lesions from PKF mice. Arrowheads indicate VCAM-1^+^ stromal cells. Scale bar, 100 µm. (**E**) Representative picture of IHC for VCAM-1 in the pancreas of wild-type mice. Scale bar, 100 µm. (**F**) Bar graph showing the result of murine VCAM-1 ELISA using supernatants of murine PDAC cell lines (K375 and K399) and cancer-associated fibroblasts (CAF) cell lines (97f. and 311f.) cultured in control media or with gemcitabine (Gem, 1 µM) for 24 h (n = 4 each). Mean ± SEM. *p < 0.05. (**G**) Bar graph showing ADAM17 activity (**H**) in murine PDAC cell lines cultured in control media or with gemcitabine (1 µM) for 24 h (n = 4 each). The result was shown as relative fluorescence unit (RFU). Mean ± SEM. *p < 0.05. (**H**) Bar graph showing relative quantification of *Adam17* mRNA expression in murine PDAC cell lines cultured in control media or with gemcitabine (1 µM) for 24 h (n = 4 each). Mean ± SEM. *p < 0.05. (**I**) Graph showing the result of ELISA for sVCAM-1 in the supernatant of K399 cells treated with TAPI-1 (50 µM) or vehicle for 1 h followed by gemcitabine at indicated concentrations (nM) for 24 h. (**J**) Bar graph showing relative quantification of *Vcam1* mRNA expression in murine PDAC cell lines cultured in control media or with gemcitabine (1 µM) for 24 h (n = 4 each). Mean ± SEM. *p < 0.05, N.S., not significant.

In search for a reliable biomarker of resistance to chemotherapy in pancreatic cancer, we utilized a genetically engineered mouse model of pancreatic cancer, *Ptf1a*^Cre/+^;Lox-STOP-Lox(LSL)-*Kras*^G12D/+^;*Tgfbr2*^flox/flox^ (PKF) mice^6^, which we generated and recapitulates human PDAC. When PKF mice were treated with gemcitabine, a standard drug used in PDAC, the overall survival was prolonged from 53 to 69 days^32^. Although it was a significant extension, tumors in this model are considered resistant to gemcitabine treatment because tumor size keeps increasing during the treatment. To understand the changes of soluble molecules in the blood in response to gemcitabine treatment, we first treated PKF mice with a single injection of gemcitabine at the age of eight weeks and collected plasma samples immediately before, 4 h after, and 48 h after the treatment. We confirmed PDAC formation in PKF mice at this age (Supplementary Fig. 1A), as previously reported^6^. Among 62 cytokines and chemokines measured by using a membrane array, sVCAM-1 was found to be one of the most increased cytokines after the treatment (Fig. 1A, Supplementary Table 1, 2). While sVCAM-1 was detected at a similar level in control wild-type (WT) mice and PKF mice before the treatment, and was not increased after the treatment in WT mice, a robust increase of sVCAM-1 was observed in PKF mice 4 h after gemcitabine treatment, and the sVCAM-1 level further increased after 48 h of the treatment (Fig. 1B).

To determine whether pancreatic cancer tissue express VCAM-1 and which type of cells express it, we immunohistochemically stained human and murine pancreatic cancer tissues for VCAM-1. In human pancreatic cancer tissues, tumor cells were found expressing VCAM-1 protein in 16 cases out of 20 cases (Fig. 1C), consistent with previous reports^30,31^. Murine PDAC tissues showed VCAM-1 expression in tumor cells, while some stromal cells also showed positive staining (Fig. 1D). PanIN2-3 lesions also showed VCAM-1 expression, and PanIN1 showed no or weak staining (Fig. 1D). VCAM-1 was not expressed in the pancreatic parenchyma of WT mice (Fig. 1E). These results indicated that VCAM-1 is expressed in cancer cells and some of the stromal cells in the pancreas, in accordance with disease progression, and sVCAM-1 is released into blood stream in response to gemcitabine treatment.

To examine whether sVCAM-1 is released from cancer cells by gemcitabine treatment, we measured sVCAM-1 protein levels in the supernatant of murine and human pancreatic cancer cell lines by enzyme-linked immunosorbent assay (ELISA). As expected, gemcitabine treatment increased sVCAM-1 in the supernatant of cancer cells (Fig. 1F, Supplementary Fig. 1B). Cancer-associated fibroblast (CAF) cell lines established from PDAC of PKF mice^33^ also showed similar results, but at a lower level (Fig. 1F). Because sVCAM-1 is known to be cleaved by an enzymatic activity of a disintegrin and metalloproteinase 17 (ADAM17)^34^, we speculated that ADAM17 activity is increased in response to gemcitabine treatment. We measured the enzymatic activity of ADAM17 in these cell lines, and found that ADAM17 activity as well as mRNA expression increased after gemcitabine treatment in cancer cell lines (Fig. 1G,H, Supplementary Fig. 1C,D).

To examine the effect of apoptosis induced by gemcitabine treatment on sVCAM-1 increase, we quantified Annexin V^+^ and Propidiun Iodide^+^ (PI^+^) cells in K399 cells treated by various concentration of gemcitabine. The result showed both Annexin V^+^ and Annexin V^+^PI^+^ cells increased with gemcitabine concentrations up to 250 nM, but these populations of cells decreased at higher doses of gemcitabine (Supplementary Fig. 1E). Cell viability assay indicated that part of K399 cells remained viable at higher concentrations of gemcitabine (Supplementary Fig. 1F), suggesting this population is resistant to gemcitabine. In ELISA assay, sVCAM1 in the media increased in a dose-dependent manner with gemcitabine concentrations up to 500 nM, but did not further increase at higher concentrations (Fig. 1I). On the other hand, when we treated K399 cells with ADAM17 inhibitor TAPI-1, sVCAM-1 in the media decreased by 62% compared to the cells without TAPI-1, and did not increase by gemcitabine treatment, suggesting the important role of ADAM17 in the increase of sVCAM-1 by gemcitabine treatment (Fig. 1I). TAPI-1 did not change viability of K399 cells (Supplementary Fig. 1G), suggesting the effect of apoptosis on the increase of sVCAM-1 in the media is relatively small.

VCAM-1 mRNA expression was modestly increased by gemcitabine treatment (24 h) in one of two murine cell lines and in a human PDAC cell line (Fig. 1J, Supplementary Fig. 1D). When we treated PKF mice with gemcitabine for 3 weeks starting from 4 weeks of age, VCAM-1 expression in PDAC tended to increase but did not reach statistical significance (Supplementary Fig. 1H).

These data suggested that ADAM17 induced sVCAM-1 release from PDAC cells in response to gemcitabine treatment, while apoptosis induced by gemcitabine may have also increased sVCAM-1.

### VCAM-1 promotes PDAC tumor growth and resistance to gemcitabine treatment in vivo

**Figure 2:**
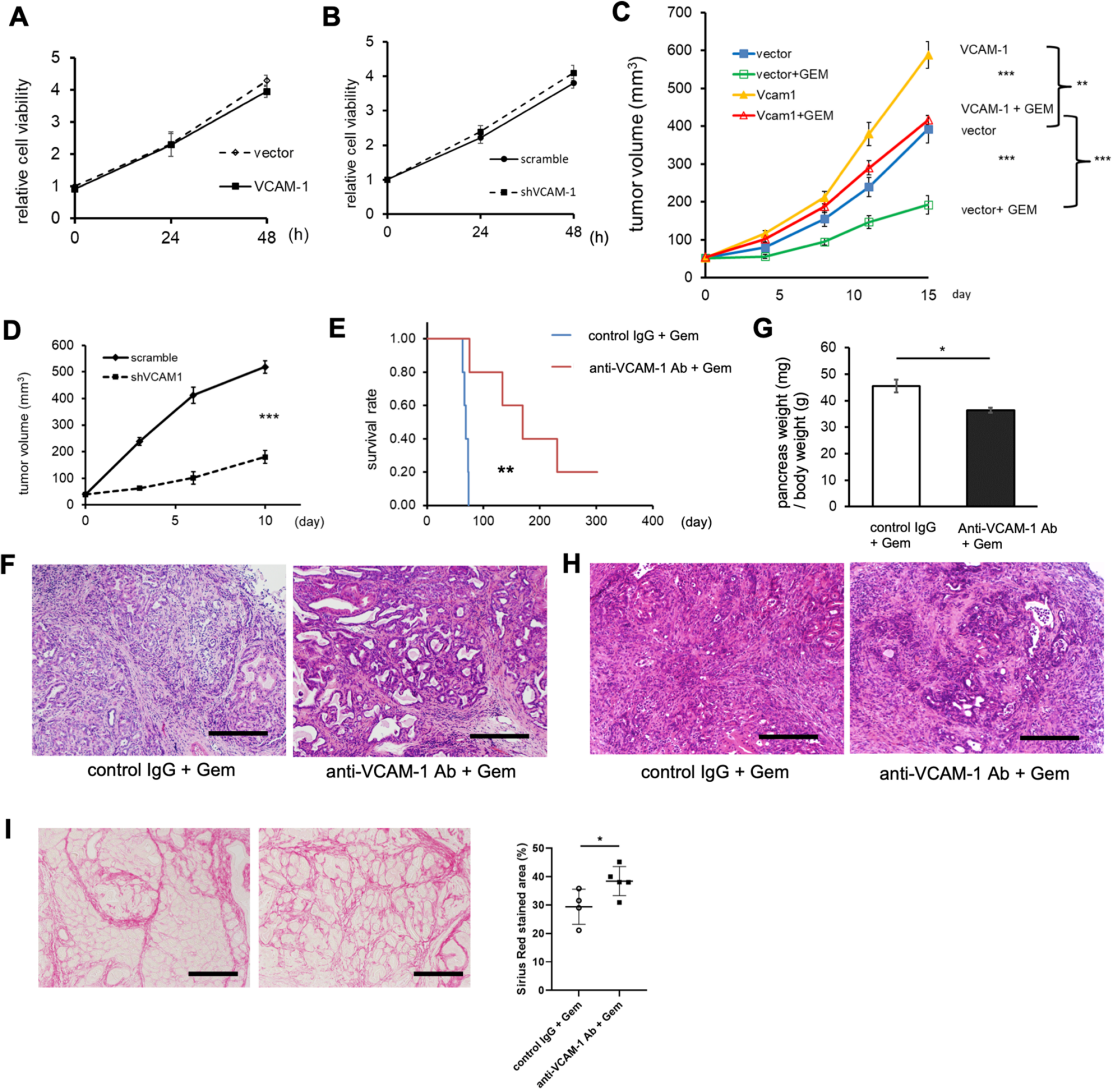
VCAM-1 induces resistance to gemcitabine treatment in PDAC. (**A**) Cell proliferation assay using K399 cell line overexpressing VCAM-1 or infected with control vector (n = 4 each). Mean ± SEM. (**B**) Cell proliferation assay using K399 cells infected with shVCAM-1 or control RNA (n = 4 each). Mean ± SEM. (**C**) Tumor volume curves of subcutaneous allografts by K399 cells overexpressing VCAM-1 or infected with control vector, treated with gemcitabine or vehicle (n = 8 each). Mean ± SEM. **p < 0.005, ***p < 0.001. (**D**) Tumor volume curves of subcutaneous allografts by K399 cells infected with shVCAM-1 or scramble shRNA (n = 8 each). Mean ± SEM. ***p < 0.001. (E) Kaplan–Meier analysis showing overall survival of PKF mice treated as shown in (**E**) (n = 5 each). **p < 0.005. (**F**) Representative pictures of H&E staining in PDAC from PKF mice treated with anti-VCAM-1 antibody (Ab) or control IgG combined with gemcitabine until they get moribund. Scale bars, 100 µm. (**G**) Bar graph showing the weight of pancreas (mg)/body weight (g) of the mice treated with anti-VCAM-1 antibody (n = 5) or control IgG (n = 4) combined with gemcitabine for 3 weeks starting from 4 weeks of age. Mean ± SEM. *p < 0.05. (**H**) Representative pictures of H&E staining in PDAC from PKF mice treated as described in (**G**). Scale bars, 100 µm. (**I**) Representative pictures of Sirius Red staining in pancreata from PKF mice treated as described in (**G**). Dot plot is showing quantitative analysis of the staining. Scale bars, 100 µm. Mean ± SD. *p < 0.05.

Next, we investigated the function of VCAM-1 in PDAC. To examine whether VCAM-1 promotes PDAC proliferation, we first evaluated the effect of VCAM-1 overexpression or knock-down on tumor growth in vitro. We generated a VCAM-1-overexpressing pancreatic cancer cell line using a PDAC cell line established from PKF mice (K399) by lentiviral infection. Overexpression of VCAM-1 mRNA and increase of sVCAM-1 protein in culture media was confirmed by qRT-PCR (Supplementary Fig. 2A) and ELISA (Supplementary Fig. 2B), respectively. We also knocked down VCAM-1 in murine pancreatic cancer cells by shRNA. Decrease of VCAM-1 expression in shVCAM-1 cells was confirmed by qRT-PCR (Supplementary Fig. 2C). Overexpression of VCAM-1 in a murine pancreatic cancer cell line did not change the proliferation of cells compared to the control vector (Fig. 2A). Similarly, knock-down of VCAM-1 in K399 did not change cell proliferation (Fig. 2B). Both suggested that sVCAM-1 is not directly affecting PDAC cells on the cell proliferation. Next, we examined the effect of sVCAM-1 in PDAC in vivo, utilizing a subcutaneous allograft model of K399 cells with VCAM-1 overexpression or knock-down (Supplementary Fig. 2D,E). Immunohistochemical staining confirmed increased or decreased expression of VCAM-1 in tumors with VCAM-1 overexpression or knock-down, respectively (Supplementary Fig. 2F,G). In contrast to the results from in vitro experiments (Fig. 2A,B), we observed VCAM-1 overexpression increased tumor volume by 48% compared to the cells with control vector (Fig. 2C). In line with this, subcutaneous allografts of the VCAM-1 knocked-down cancer cells showed delayed tumor growth compared to cancer cells with scramble shRNA (Fig. 2D). These results suggested interactions of VCAM-1 with host environment, rather than direct effects on cancer cells, which accelerate the tumor growth.

To see the effect of VCAM-1 on gemcitabine resistance in pancreatic cancer in vitro, we treated K399 cells with VCAM-1 overexpression or knock-down with gemcitabine. As a result, VCAM-1 overexpression or knock-down did not clearly affect resistance to gemcitabine (Supplementary Fig. 2H). To examine whether sVCAM-1 promotes gemcitabine resistance in pancreatic cancer in vivo, we treated VCAM-1-overexpressing allograft tumors or control tumors with gemcitabine. While both groups showed reduction of tumor volume compared to the tumors without gemcitabine, VCAM-1-overexpressing tumors showed significantly less reduction of tumor volume compared to that of control tumors (30% vs 51% reduction, respectively) after gemcitabine treatment (Fig. 2C). Similarly, when we calculated tumor growth rate over day 11 to day 15 and compared the reduction of tumor growth rate, we observed less reduction of tumor growth rate in VCAM-1-overexpressing tumors compared to control tumors (38.4% vs 71.3% reduction, respectively) (Supplementary Fig. 2I).

To further examine the effect of sVCAM-1 on gemcitabine resistance in vivo, we treated PKF mice with VCAM-1 neutralizing antibody or control IgG in combination with gemcitabine. Overall survival of the mice was dramatically prolonged in antibody-treated mice (median survival time, MST: 170 days) compared to the control mice (MST: 69 days, *p* < 0.05) (Fig. 2E), although we found PDAC in both groups (Fig. 2F). Next, we treated PKF mice with gemcitabine combined with anti-VCAM-1 antibody or control IgG for 3 weeks starting from 4 weeks of age. At 4 weeks of age, PDAC was found in one out of four mice and PanIN2-3 lesions were found in three out of four mice (Supplementary Fig. 2J). The weight of the pancreas significantly decreased in antibody-treated mice compared to the control mice (Fig. 2G), while we observed PDAC in both groups (Fig. 2H). Immunohistological analysis showed tendency to decreased F4/80^+^ cells and Ki67^+^ cells, although not significant. Desmoplastic area measured by Sirius red staining in the tumor significantly increased and cleaved caspase3^+^ apoptotic cells tended to increase by anti-VCAM-1 antibody (Fig. 2I, Supplementary Fig. 2K). These results suggested that sVCAM-1 promoted the resistance to gemcitabine treatment in vivo, and blocking VCAM-1 may increase sensitivity to gemcitabine in PDAC.

### Soluble VCAM-1 attracts macrophages to PDAC microenvironment to promote gemcitabine resistance

Next, we examined the mechanism how sVCAM-1 promotes resistance to gemcitabine in PDAC. Because sVCAM-1 is reported to attract monocytic cells in various diseases such as rheumatoid arthritis^35^ and breast cancer^20^, and it was reported that macrophages induce therapy resistance in PDAC^36^, we hypothesized that sVCAM-1 has chemotactic effects on macrophages towards PDAC microenvironment to induce resistance to gemcitabine.

**Figure 3:**
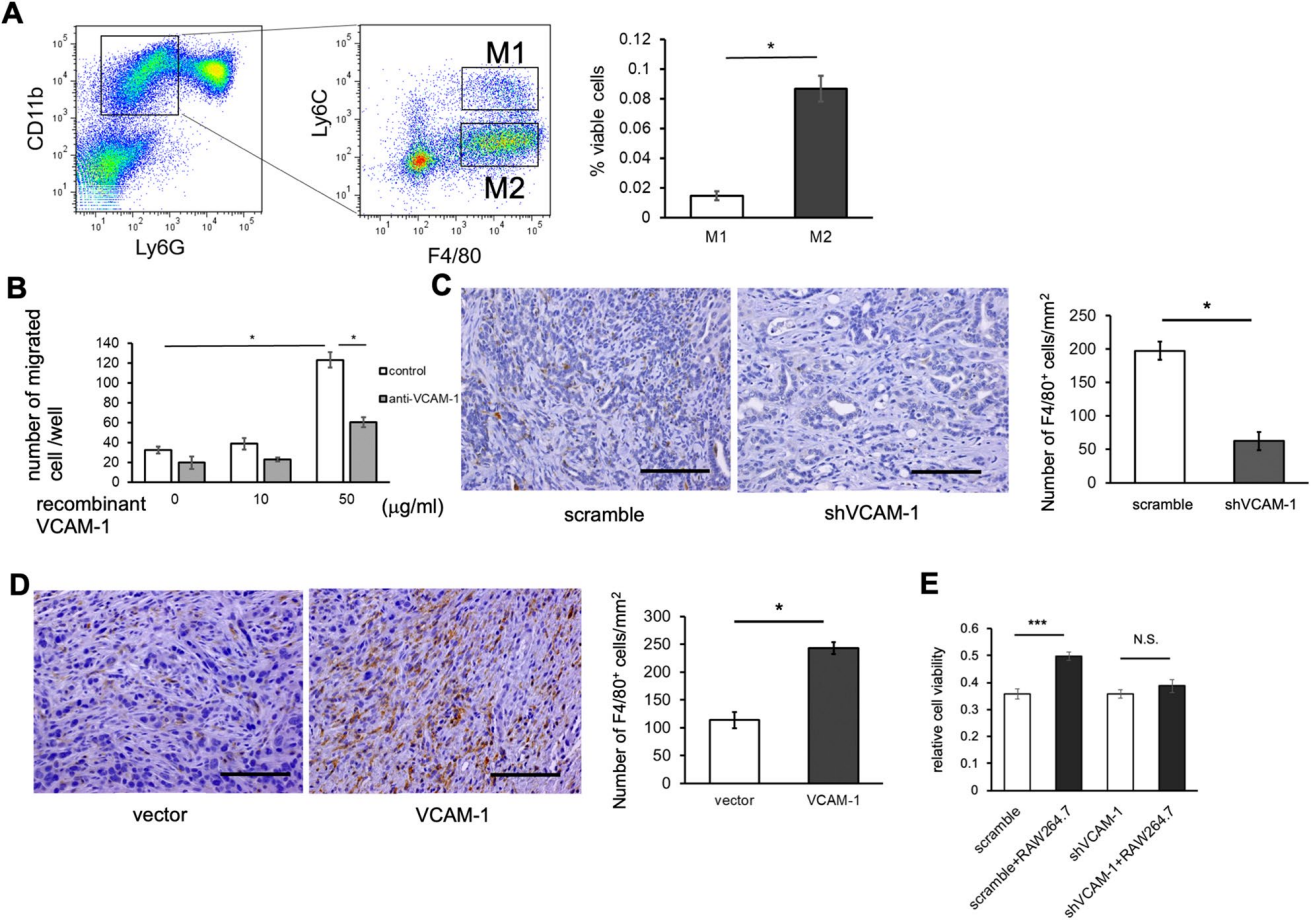
Tumor-associated macrophages are attracted by soluble VCAM-1 to promote gemcitabine resistance. (**A**) Flow cytometric analysis of macrophage population within PDAC of PKF mice. Bar graph is showing ratio of M1 and M2 macrophages among living single cells from the PDAC tissue (n = 4 each). Mean ± SEM. *p < 0.05. (**B**) Bar graph showing the result of migration assay, measuring the number of migrated RAW264.7 cells in the presence of recombinant murine VCAM-1 at indicated concentrations with or without anti-VCAM-1 antibody (10 µg/ml) (n = 4 each). Mean ± SEM. *p < 0.05. (**C**) Representative images of IHC for F4/80 in subcutaneous allografts by K399 cells infected with shVCAM-1 or scramble shRNA. Bar graph is showing quantitative analysis of F4/80^+^ cells (n = 8 each). Mean ± SEM. *p < 0.05. Scale Bars, 50 μm. (**D**) Representative images of IHC for F4/80 in subcutaneous allografts by K399 cells overexpressing VCAM-1 or infected with control vector. Bar graph is showing quantitative analysis of F4/80^+^ cells (n = 8 each). Mean ± SEM. *p < 0.05. Scale Bars, 50 μm. (**E**) Bar graphs showing the result of cell proliferation assay using K399 cells infected with shVcam-1 or scramble shRNA, cultured with or without RAW264.7 and treated with gemcitabine (1 µM) for 24 h (n = 4 each). Mean ± SEM. ***p < 0.001. NS, not significant.

In immunohistochemistry, we observed abundant infiltration of F4/80^+^ macrophages in the stromal area of PDAC in PKF mice (Supplementary Fig. 3A), as previously reported^6^. By flow cytometric analysis, we found these macrophages are skewed into M2 tumor-associated macrophages (TAMs)^37^ (Fig. 3A), recapitulating human PDAC^38^. To see whether macrophages are attracted by sVCAM-1 in vitro, we cultured RAW264.7 cells, a murine macrophage cell line, in a culture insert with recombinant VCAM-1 in the bottom chamber. RAW264.7 cells showed increased migration toward the chamber containing recombinant VCAM-1 (Fig. 3B) and the migration was blocked when the cells were cultured with VCAM-1 neutralizing antibody. To examine whether sVCAM-1 attracts macrophages to PDAC microenvironment in vivo, we evaluated F4/80^+^ macrophages in subcutaneous allograft tumors from PDAC cells with shVCAM-1. As expected, VCAM-1 knocked-down tumors showed less infiltration of F4/80^+^ macrophages compared to tumors established from the control cells (Fig. 3C). In contrast, we observed increased infiltration of macrophages in allograft tumors from VCAM-1-overexpressing PDAC cells (Fig. 3D). These results suggested that sVCAM-1 attracts macrophages to PDAC microenvironment.

To examine whether increased sVCAM-1 and macrophages in the tumor microenvironment induce gemcitabine resistance in PDAC cells, we treated PDAC cells by gemcitabine and analyzed the response. In a monoculture setting, knock-down of VCAM-1 alone did not change cell viability against gemcitabine treatment (Figs. 2B, 3E, and Supplementary Fig. 2H). In addition, culturing PDAC cells with recombinant VCAM-1 did not increase viability of the cells after gemcitabine treatment (data not shown). These results suggested that sVCAM-1 does not directly induce gemcitabine resistance in PDAC cells. In contrast, when we co-cultured K399 cells with RAW264.7 cells in a two-chamber setting and treated them with gemcitabine, we observed increased viability of PDAC cells after gemcitabine treatment (Fig. 3E, Supplementary Fig. 3B) compared to K399 alone, although GI50 showed only a small difference. This effect was cancelled when we used cancer cells with VCAM-1 knocked down (Fig. 3E, Supplementary Fig. 3B). Collectively, these results suggested that sVCAM-1 secreted from PDAC cells attracts macrophages to the tumor microenvironment, thereby inducing resistance to gemcitabine treatment.

### Changes in soluble VCAM-1 level in the plasma of PDAC patients is an independent prognostic factor for gemcitabine treatment

**Figure 4:**
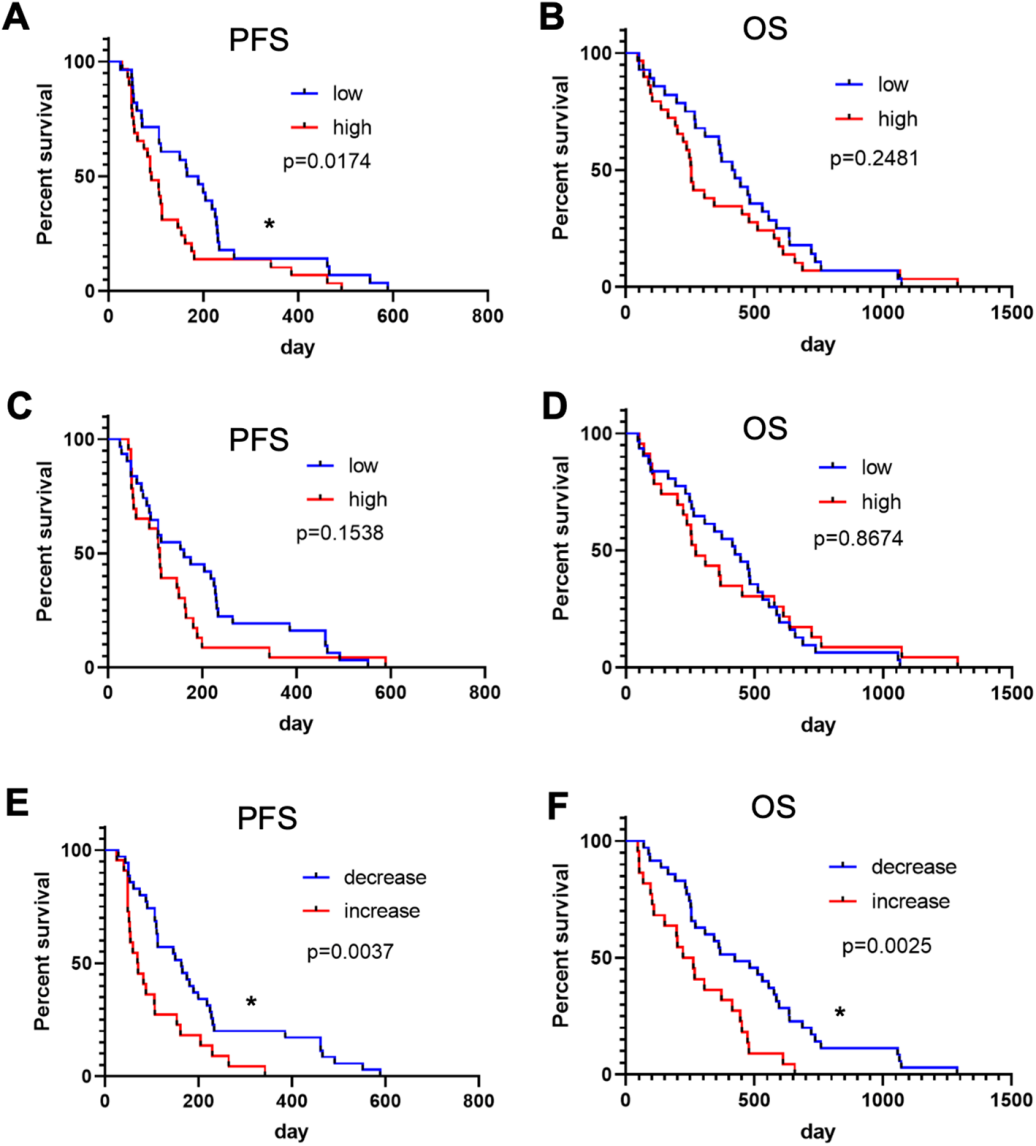
Changes in plasma soluble VCAM-1 level is correlated to survival of PDAC patients. (**A**–**B**) Kaplan–Meier Curves showing PFS (**A**) and OS (**B**) of patients with lower or higher plasma CA19-9 levels at the beginning of gemcitabine treatment (n = 28 each). Median PFS was 199 and 90 days, and median OS was 445 and 255 days in patients with lower or higher CA19-9 level, respectively. *p < 0.05. (**C**–**D**) Kaplan–Meier Curves showing PFS (**C**) and OS (**D**) of patients with lower or higher soluble VCAM-1 levels in the plasma at the beginning of gemcitabine treatment (n = 28 each). Median PFS was 161 and 109 days, and median OS was 424 and 271 days in patients with lower or higher VCAM-1 level, respectively. (**E**–**F**) Kaplan–Meier Curves showing PFS (**E**) and OS (**F**) of patients with decreased (n = 35) or increased (n = 22) soluble VCAM-1 levels in the plasma during the first four weeks of gemcitabine treatment. Median PFS was 163 and 82 days, and median OS was 424 and 263 days in patients with decreased or increased VCAM-1 level, respectively. *p < 0.05.

These experimental results prompted us to investigate whether the level of sVCAM-1 in the plasma of pancreatic cancer patients might be correlated with response to gemcitabine treatment, serving as a clinical marker for the therapy response. To explore this possibility, we examined the association of sVCAM-1 levels detected in the plasma and the response to gemcitabine treatment in a clinical setting. We measured sVCAM-1 levels in human plasma samples of the patients with unresectable pancreatic cancer (n = 57) who received gemcitabine treatment as the first-line chemotherapy. Patient characteristics are shown in Table 1. Median progression-free survival (PFS) in this cohort was 110 days, and median overall survival (OS) was 344 days. We measured sVCAM-1 in the plasma immediately before the first treatment with gemcitabine and four weeks after the treatment. CA19-9 was also measured at the same time points. In a correlation analysis, Spearman’s coefficiency was 0.047, suggesting that sVCAM-1 level has only a weak correlation with CA19-9 level (Supplementary Fig. 4A). In addition, the level of sVCAM-1 did not show significant difference between patients with metastasis and locally advanced disease (Supplementary Fig. 4B). When patients were separated into two groups according to their CA19-9 levels at diagnosis, patients with CA19-9 level below the median (992 U/ml) showed significantly longer PFS (199 days) compared to those above the median (90 days) (Fig. 4A), as previously reported^39^, although OS was not significantly different in these groups (445 days and 255 days) (Fig. 4B). When the patients were categorized by sVCAM-1 levels before treatment, patients with lower VCAM-1 level below the median (346.0 ng/ml) showed a tendency of longer median PFS (161 days) and OS (424 days) compared to those with higher VCAM-1 level (PFS 109 days, OS 263 days), although it was not statistically significant (Fig. 4C,D). In contrast, when patients were categorized by the change of sVCAM-1 level after four weeks of treatment, patients with a decrease of VCAM-1 level (n = 35) showed significantly longer PFS (163 days) and OS (424 days) than the patients with an increase of sVCAM-1 level (n = 22) (PFS 70 days and OS 222 days) (Fig. 4E,F). Change in CA19-9 was similarly analyzed to compare with sVCAM-1 change, and the result indicated that it was significantly correlated with OS and tended to correlate with PFS (Supplementary Fig. 4C). To determine an impact of the “VCAM-1 response” on the prognosis of unresectable pancreatic cancer, the Cox proportional hazard model was used, and univariate and multivariate analyses were performed for the PFS and OS (Tables 2 and 3). VCAM-1 response (decrease in VCAM-1 level) was prognostic for both PFS (*p* = 0.006) and OS (*p* = 0.004) in the univariate analysis. In multivariate analysis, lack of metastasis was the most significantly associated with longer PFS (*p* = 0.036) and OS (*p* = 0.008). VCAM-1 response was also independently associated with better PFS (*p* = 0.046), but not with OS (*p* = 0.062). These data suggested the changes in sVCAM-1 level in the plasma of patients is an independent prognostic factor for gemcitabine treatment, which may reflect the resistance of cancer cells and help predicting efficacy of gemcitabine treatment as early as one month after the induction of treatment.

**Table 1:** Summary of patient characteristics.

	VCAM-1 decrease (n = 35)	VCAM-1 increase (n = 22)	*p* value
Age	65 (47–84)	63 (40–83)	0.137
Sex: male/female	23 (66%)/12 (34%)	13 (59%)/9 (41%)	0.779
**Stage**			0.150
Locally advanced	13 (37%)	4 (18%)	
Metastatic	22 (63%)	18 (82%)	
PS: 0/1–2	21 (60%)/14 (40%)	11 (50%)/ll (50%)	0.585
CA19-9, U/ml	962 (1–109,250)	1328(1–168,200)	0.831
VCAM-1, ng/ml	346.0(123.4–739.1)	313.8 (71.6–926.0)	0.31

Numbers are shown either as absolute numbers (%) or median (range).

*PS* performance status.

**Table 2:** Prognostic factors for PFS.

	Univariate analysis HR (95%CI)	*p* value	Multivariate analysis HR (95%CI)	*p* value
Age: ≥ 65	1.01 (0.59–1.72)	0.963		
Sex: Male	1.13 (0.66–1.99)	0.652		
Stage: Metastatic	2.36 (1.30–4.55)	0.004	1.98 (1.05–3.92)	0.036
PS: 1–2	1.09 (0.63–1.86)	0.760		
Ln (CA19-9)*	1.15 (1.01–1.32)	0.037	1.12 (0.99–1.27)	0.081
Ln (VCAM-1)*	1.55 (0.77–3.15)	0.219		
VCAM-1 increase	2.25 (1.27–3.96)	0.006	1.83 (1.01–3.28)	0.046

*HR* hazard ratio.

*HR per 1 increase.

**Table 3:** Prognostic factors for OS.

	Univariate analysis HR (95%CI)	*p* value	Multivariate analysis HR(95%CI)	*p* value
Age ≥ 65	1.07 (0.63–1.83)	0.794		
Sex: Male	1.00 (0.58–1.75)	1.000		
Stage: Metastatic	3.12 (1.65–6.27)	< 0.001	2.57 (1.305]^V^-S.34)	0.008
PS: 1–2	1.25 (0.72–2.12)	0.424		
Ln (CA19-9)*	1.11(0.97–1.27)	0.130	1.05 (0.92–1.20)	0.482
Ln (VCAM-1)*	1.05 (0.61–1.86)	0.868		
VCAM-1 increase	2.39 (1.33–4.26)	0.004	1.77 (0.97–3.22)	0.062

*HR* hazard ratio.

*HR per 1 increase.

## Discussion

While it was reported that VCAM-1 expression in surgically resected PDAC tissue was associated with patient prognosis^31^, VCAM-1 expression in tumor tissue is unknown in advanced pancreatic cancer. The role of sVCAM-1 in PDAC has not been clear. In this study, we showed an important role of sVCAM-1 in resistance to gemcitabine treatment in PDAC. Furthermore, we have shown the association of sVCAM-1 level in the plasma of PDAC patients with therapy response. Given that the majority of PDAC patients are ineligible for surgical resection and undergo chemotherapy including gemcitabine, our finding may be applicable to most of PDAC cases.

While we showed VCAM-1 was expressed in the cancer cells in pancreatic cancer tissue by immunohistochemistry, some of the stromal cells were observed expressing VCAM-1. Thus, there are potentially multiple cell components secreting sVCAM-1 in PDAC microenvironment. It should be determined whether sVCAM-1 from stromal cells has similar function shown in our study. Nevertheless, we showed CAF cell line secreted sVCAM-1 at a lower level compared to cancer cells, suggesting that sVCAM-1 is mainly shed from cancer cells.

Expression of VCAM-1 has been reported to be regulated by multiple signaling pathways including nuclear factor kappa B (NF-κB) and phosphatidylinositol 3-kinase pathways^40,41^. It was reported that these pathways were activated by gemcitabine treatment in pancreatic cancer cells and induced resistance to the treatment^42,43^. While our data showing upregulation of VCAM-1 by gemcitabine treatment was in line with these reports, our results also suggested an additional mechanism involving sVCAM-1 and macrophages. It should be determined whether other chemokines which attract macrophages, such as monocyte chemotactic protein 1, are also upregulated by gemcitabine treatment.

Macrophages infiltrated in the tumor have been suggested to induce immune suppression and resistance to chemotherapy^36^. Our data suggested that sVCAM-1 attracts macrophages to pancreatic cancer microenvironment and induces resistance to gemcitabine treatment. In particular, the effect of VCAM-1 antibody treatment clearly increased the efficacy of gemcitabine treatment. Although it is possible that immunodeficiency in the mice might have affected the allograft study, results observed in PKF mice treated with anti-VCAM-1 antibody suggested that immunocompetent mice have similar responses in terms of sVCAM-1 mediated resistance to gemcitabine. Given that currently there is no established therapy to inhibit gemcitabine resistance, targeting sVCAM-1 might be a therapeutic option to overcome therapy resistance. Further investigation is needed to understand detailed underlying mechanisms of gemcitabine resistance induced by infiltrated macrophages.

We observed differences in the effect of VCAM-1 expression on tumor growth and gemcitabine resistance between in vitro 2D and in vivo 3D models. While tumor cell growth was not affected by VCAM-1 in 2D culture, we observed increased tumor growth in allograft models. These results suggested that the mechanism of increased tumor growth by VCAM-1 may depend on 3D condition or interaction with host cells. On the other hand, we observed increased resistance to gemcitabine treatment both in vitro 2D co-culture with RAW264.7 cells and in vivo 3D condition, which suggested that VCAM-1 could induce gemcitabine resistance by interaction with host cells.

While we have shown prolonged survival in PKF mice treated with gemcitabine and anti-VCAM-1 antibody compared to control IgG, there are some limitations. First, it should be noted that PKF mice at 4 weeks of age develop high-grade PanIN but PDAC is uncommon, which suggests that the prolonged survival and decreased pancreas weight may be affected by delayed onset of PDAC formation. Second, given that VCAM-1 seemed to have effects on tumor growth and gemcitabine resistance in allograft models, anti-VCAM-1 antibody monotherapy may also have tumor inhibitory effects. As we did not have anti-VCAM-1 antibody monotherapy group, the effect of the antibody on tumor growth and gemcitabine resistance could not be assessed separately in this study. Further experiments are needed to determine whether VCAM-1 inhibition prolongs survival of mice with PDAC, and delays tumor development in PKF mice. In addition, investigating the underlying mechanism would be needed to fully understand the role of VCAM-1 in PDAC development and progression.

In a clinical setting, we have shown that changes in plasma sVCAM-1 is correlated to response to gemcitabine and prognosis in advanced PDAC patients. While CA19-9 is a widely used tumor marker of pancreatic cancer, early responses in CA19-9 as a prognosis marker has been controversial^44–46^. In line with a previous report^46^, our data suggested that CA19-9 change might be correlated with patient prognosis. However, in our data set, it did not show a better correlation compared to sVCAM-1 change. In addition, CA19-9 is not useful for patients who lack Lewis Blood group phenotype^47^. In this study, our results suggested that changes in sVCAM-1 might be a marker of response to gemcitabine, including for those who lack CA19-9, although further validation would be necessary in future studies.

In contrast to a previous study^31^, we have analyzed soluble form of VCAM-1 in advanced diseases, in which tissue samples are mostly unavailable. Measurement of sVCAM-1 in the plasma can be longitudinally repeated during chemotherapy. Our observation that the change of sVCAM-1 was correlated with PFS but not with OS in multivariate analysis suggests that evaluation of therapy resistance is necessary for each subsequent therapy. It remains to be determined whether sVCAM-1 serves as a marker of therapy response for other chemotherapies, such as gemcitabine plus nab-paclitaxel and FOLFIRINOX^48,49^. Previous studies indicated that NF-κB pathway is also involved in resistance to platinum agent^50^, which may suggest that sVCAM-1 is involved in resistance to these therapies as well.

In conclusion, we have shown that sVCAM-1 is elevated in PDAC in response to gemcitabine treatment, which attracts macrophages to PDAC microenvironment and induces resistance to gemcitabine treatment. Blocking sVCAM-1 enhanced the efficacy of gemcitabine and prolonged the survival of PDAC mice. In addition, sVCAM-1 in the blood may be a clinical marker to indicate gemcitabine response in PDAC patients. These results suggested sVCAM-1 is a potential therapeutic target, as well as a prognosis marker, in advanced PDAC patients.

## Methods

### Mouse models

*Ptf1a*^cre/+^;LSL-*Kras*^G12D/+^;*Tgfbr2*^flox/flox^ mice (PKF mice) and *Ptf1a*^cre/+^;LSL-*Kras*^G12D/+^ mice were described previously^6^. These genetically engineered mice and wild-type control (Clea-Japan, Tokyo, Japan) were with C57BL/6 background. BALB/cAjcl-*nu/nu* mice (Clea-Japan) were used for allograft experiments. All animal experimental protocols were approved by the Ethics Committee for Animal Experiments of the University of Tokyo and performed with adherence to ARRIVE (Animal Research: Reporting of In Vivo Experiments) guidelines (https://www.nc3rs.org.uk/arrive-guidelines). All animals were kept in specific pathogen-free housing with abundant standard diet and water. Interventions were performed during light cycle.

### Human tissue and plasma samples

Human pancreatic cancer tissue array was purchased from US Biomax (Derwood, MD). Plasma samples of pancreatic cancer patients treated with gemcitabine monotherapy were obtained at the University of Tokyo hospital. All samples were used anonymized. This study was approved by the Ethics Committee of the University of Tokyo, and carried out in accordance with the Declaration of Helsinki. Informed consent in writing was obtained from all patients.

### Cell culture

All human pancreatic cancer cell lines and RAW264.7 cells were purchased from ATCC. Murine pancreatic cancer cell lines (K375 and K399) and cancer associated fibroblast (CAF) cell lines (97f. and 311f.) were derived from PKF mice^6,33,51,52^. All cell lines were cultured in RPMI-1640 medium supplemented with 10% FBS in a humidified incubator at 37 °C. Cells were treated with gemcitabine (Yakult, Tokyo, Japan) and/or TAPI-1 (Cayman Chemical, Ann Arbor, MI, USA) for further experiments.

### Mice treatment and plasma collection

PKF and wild-type mice at 8 weeks old were injected with 12.5 mg/kg gemcitabine intraperitoneally. Plasma was collected by cardiac puncture before gemcitabine treatment as well as 4 h and 48 h after gemcitabine treatment, respectively. Four mice in each group were enrolled and the plasma samples in the same group were pooled, then used for cytokine array.

For VCAM-1 neutralizing antibody treatment in combination with gemcitabine, PKF mice were i.p. injected with 50 µg rat anti-mouse VCAM-1 (CD106; IgG1 kappa) antibody (SouthernBiotech, Birmingham, AL, USA) or 50 µg purified rat IgG1 kappa isotype control (BioLegend, Tokyo, Japan) five times a week, respectively, and both groups were also i.p. injected with 12.5 mg/kg gemcitabine twice a week. The injection was started at 4 weeks of age and continued until the mice get moribund (n = 5) or for 3 weeks (n = 4 in control IgG group and n = 5 in anti-VCAM-1 antibody group).

### Cytokine array

Membrane-based cytokine array (RayBiotech, Peachtree Corners, GA, USA) was performed using murine plasma according to manufacturer’s protocol. The full list of cytokines measured is shown in Supplementary Table1. The density of each spot was determined using ImageJ software. The quantification data was shown in Supplementary Table 2.

### Histology and immunohistochemistry

Murine tissues were harvested and processed as described before^6^. Primary antibodies used were anti-VCAM-1 antibody (Santa Cruz Biotechnology, Dallas, TX), anti-F4/80 antibody (Bio-Rad, Hercules, CA), anti-Ki67 antibody (Abcam, Cambridge, United Kingdom) and anti-cleaved caspase3 antibody (Cell Signaling Technologies, Danvers, MA, USA). Antigen retrieval was performed by boiling the slides in citrate buffer (10 mM, pH 6.0) in a water bath for 20 min. For immunohistochemistry, tissue arrays were processed and stained similarly. For Sirius red staining, formalin-fixed paraffin-embedded sections were deparaffinized, rehydrated, and stained for 10 min in 0.1% Sirius red in saturated picric acid (Wako Pure Chemical Insudtries, Osaka, Japan).

### ELISA

VCAM-1 ELISA assay for murine and human plasma samples and cell culture supernatant were performed using DuoSet ELISA Development kit (R&D Systems, Minneapolis, MN, USA) and Mouse VCAM-1 ELISA kit (Abcam) according to the manufacturer’s protocol.

### TACE activity assay

Activity of ADAM-17 was determined using SensoLyte 520 TACE Activity Assay Kit (AnaSpec, Fremont, CA) according to the manufacturer’s protocol and analyzed by fluorescent microplate reader (BioTek, Winooski, VT).

### Quantitative RT-PCR (qRT-PCR)

Total RNA was extracted from cell cultures using RNA*later* stabilization solution (Thermo Fisher Scientific, Walthum, MA) and NucleoSpin RNA II (Machery-Nagel, Düren, Germany) and subjected to first-strand complementary DNA synthesis using the ImProm-II Reverse Transcription System (Promega, Madison, WI). qRT-PCR was performed on the StepOnePlus Real-Time PCR System (Thermo Fisher Scientific) using the FastStart Universal SYBR Green Master (Roche, Basel, Switzerland). Each assay was performed using biological samples in triplicate. Relative expression was calculated as a ratio of each gene expression to that of *Actb* or *ACTB*. The sequences of the primers used are shown in Supplementary Table 3.

### Migration assay and coculture assay

For migration assay using RAW264.7 cells, 10^5^ cells were seeded in cell culture inserts with 8 μm pores, and 0, 10 and 50 µg/ml of recombinant VCAM-1 was added into the media in the bottom well. After incubating 10 h with or without anti-VCAM-1 neutralizing antibody (Millipore, Burlington, MA), migrated cells were stained by Diff-Quik staining (Sysmex, Kobe, Japan).

For coculture of RAW264.7 cells and PDAC cells, 1 × 10^4^ RAW264.7 cells were seeded in cell culture inserts with 0.4 μm pores and 5 × 10^4^ PDAC cells were seeded in the bottom wells. After incubating with gemcitabine for 24 h, CCK-8 (Dojindo, Kumamoto, Japan) was used to measure cell viability.

### Flow cytometry analysis

Murine pancreatic tumor was harvested and chopped into small pieces, and digested in 0.25% Trypsin EDTA (Thermo Fisher Scientific) at 37 °C for 15 min. After neutralizing with fetal bovine serum (FBS), samples were further digested with 2 mg/ml collagenase A (Roche) and DNase I (50 U/ml, Worthington, Columbus, OH) at 37 °C for 20 min. Following multiple washes with PBS supplemented with 2% FBS, cells were filtered through a 40 µm cell strainer. Red blood cells were lysed with ACK Lysing Buffer (Thermo Fisher Scientific) after passing through a strainer. Filtered single cells were incubated with anti-CD11b (Thermo Fisher Scientific), F4/80, Ly6C and Ly6G antibodies (Biolegend, San Diego, CA). LIVE/DEAD Fixable Dead Cell Stain Kit (Thermo Fisher Scientific) was used to exclude dead cells. Cells were analyzed by BD FACSAria II (BD Biosciences, Franklin Lakes, NJ). For cell apoptosis assay, MEBCYTO apoptosis kit (Medical Biological Laboratories, Nagoya, Japan) was used according to manufacturer’s protocol and analyzed using Guava easyCyte plus (Luminex, Austin, TX, USA).

### Short hairpin RNAs

Knock-down experiments were performed as described previously^52^ using pLKO.1-puro-based lentiviruses expressing specific short hairpin RNAs (shRNAs) following the manufacturer’s instruction (Addgene, Watertown, MA). We infected indicated cells with lentiviruses expressing shRNAs in the presence of 8 μg/ml polybrene. After 24 h, stably transfected cells were selected with puromycin (4 µg/ml). A scramble shRNA, used as a negative control, and shRNA against VCAM-1 were purchased from Open Biosystems (Horizon Discovery, Cambridge, United Kingdom).

### VCAM-1-expressing cells

To produce VCAM-1-expressing cells, we cloned murine VCAM-1 cDNA into pLVSIN-EF1α Pur vector (Takara Bio). Lentiviral particles were obtained as described previously^52^ and infected into K399 cells. After selected by puromycin, polyclonal cells were used as VCAM-1-expressing cells. Lentivirus carrying empty vectors were used as control.

### Cell viability assay

In vitro cell viability assay was performed using Cell Counting Kit-8 (Dojindo, Tokyo, Japan) according to the manufacturer’s instructions. Briefly, cells were plated in triplicate in 48-well dishes. On the next day evaluation of cell proliferation was started, and the evaluation was performed at 0–48 h. For the last 2 h of incubation, cells were pulsed with 10 µl CCK8 reagent (Dojindo) into 100 µl of culture media, and then absorbance of 450 nm was measured.

### Allograft models

3 × 10^6^ K399 cells with VCAM-1 knock-down or overexpression as well as control cells of each were injected subcutaneously into the flank of BALB/cAjcl-*nu/nu* mice (female, 6 weeks old). One week after injection, we started measuring tumor volume. Tumor volume was calculated by 0.5 × (long diameter) × (short diameter)^2^. Then mice were treated with 100 mg/kg gemcitabine or vehicle intraperitoneally twice a week. Each group contained 16 tumors.

### Statistical analysis

Differences between means were compared by using Student’s *t-*test or Wilcoxon test. The survival curves were plotted according to the Kaplan–Meier method and compared by log-rank test. Spearman’s correlation analysis was performed for correlation of two parameters. The Cox proportional hazards model was used to analyze the prognostic factors for PFS and OS. The Cox model analyses included age (< 65 vs. ≥ 65 years), sex, stage (locally advanced vs. metastatic), WHO performance status (PS: 0 vs. ≥ 1), CA19-9, VCAM-1, and the VCAM-1 response. Natural logarithm of CA19-9 and VCAM-1 value was used because these variables were not following a normal distribution. Prognostic factors with a *p* value < 0.2 in univariate analysis were evaluated using Fisher’s exact test. All reported *p* values are the results of two-sided tests, with *p* < 0.05 considered statistically significant.

## Supplementary information

Supplementary information.

## Data Availability

The data that support the findings of this study are available from the corresponding author on reasonable request.

## References

[1] Kato, K. Vital Statistics in Japan. Director-General for statistics and Information Policy, Ministry of Health, Labour and Welfare: Tokyo, 18–19 (2017).

[2] Rahib L (2014). Projecting cancer incidence and deaths to 2030: the unexpected burden of thyroid, liver, and pancreas cancers in the United States. Cancer Res..

[3] Siegel RL, Miller KD, Jemal A (2019). Cancer statistics, 2019. Cancer J. Clin..

[4] Ryan DP, Hong TS, Bardeesy N (2014). Pancreatic adenocarcinoma. N. Engl. J. Med..

[5] Ballehaninna UK, Chamberlain RS (2011). Serum CA 19–9 as a biomarker for pancreatic cancer-a comprehensive review. Indian J. Surg. Oncol..

[6] Ijichi H (2006). Aggressive pancreatic ductal adenocarcinoma in mice caused by pancreas-specific blockade of transforming growth factor-beta signaling in cooperation with active Kras expression. Genes Dev..

[7] Aguirre AJ (2003). Activated Kras and Ink4a/Arf deficiency cooperate to produce metastatic pancreatic ductal adenocarcinoma. Genes Dev..

[8] Hingorani SR (2003). Preinvasive and invasive ductal pancreatic cancer and its early detection in the mouse. Cancer Cell.

[9] Hingorani SR (2005). Trp53R172H and KrasG12D cooperate to promote chromosomal instability and widely metastatic pancreatic ductal adenocarcinoma in mice. Cancer Cell.

[10] Bardeesy N (2006). Both p16(Ink4a) and the p19(Arf)-p53 pathway constrain progression of pancreatic adenocarcinoma in the mouse. Proc. Natl. Acad. Sci. U S A.

[11] Olive KP (2009). Inhibition of Hedgehog signaling enhances delivery of chemotherapy in a mouse model of pancreatic cancer. Science.

[12] Singh M (2010). Assessing therapeutic responses in Kras mutant cancers using genetically engineered mouse models. Nat. Biotechnol..

[13] Wittchen ES (2009). Endothelial signaling in paracellular and transcellular leukocyte transmigration. Frontiers Biosci..

[14] Ley K, Laudanna C, Cybulsky MI, Nourshargh S (2007). Getting to the site of inflammation: the leukocyte adhesion cascade updated. Nat. Rev. Immunol..

[15] Wieland E (2017). Endothelial Notch1 Activity Facilitates Metastasis. Cancer Cell.

[16] Chen Q, Zhang XH, Massague J (2011). Macrophage binding to receptor VCAM-1 transmits survival signals in breast cancer cells that invade the lungs. Cancer Cell.

[17] Lin KY (2007). Ectopic expression of vascular cell adhesion molecule-1 as a new mechanism for tumor immune evasion. Cancer Res..

[18] Kuai WX (2012). Interleukin-8 associates with adhesion, migration, invasion and chemosensitivity of human gastric cancer cells. World J. Gastroenterol..

[19] Ding YB (2003). Association of VCAM-1 overexpression with oncogenesis, tumor angiogenesis and metastasis of gastric carcinoma. World J. Gastroenterol..

[20] Lu X (2011). VCAM-1 promotes osteolytic expansion of indolent bone micrometastasis of breast cancer by engaging alpha4beta1-positive osteoclast progenitors. Cancer Cell.

[21] Alexiou D (2001). Serum levels of E-selectin, ICAM-1 and VCAM-1 in colorectal cancer patients: correlations with clinicopathological features, patient survival and tumour surgery. Eur. J. Cancer.

[22] Dymicka-Piekarska V, Guzinska-Ustymowicz K, Kuklinski A, Kemona H (2012). Prognostic significance of adhesion molecules (sICAM-1, sVCAM-1) and VEGF in colorectal cancer patients. Thromb Res..

[23] Velikova G (1997). Circulating soluble adhesion molecules E-cadherin, E-selectin, intercellular adhesion molecule-1 (ICAM-1) and vascular cell adhesion molecule-1 (VCAM-1) in patients with gastric cancer. Br. J. Cancer.

[24] Shioi K (2006). Vascular cell adhesion molecule 1 predicts cancer-free survival in clear cell renal carcinoma patients. Clin. Cancer Res..

[25] van der Veldt AA (2012). Sunitinib-induced changes in circulating endothelial cell-related proteins in patients with metastatic renal cell cancer. Int. J. Cancer.

[26] Huang J (2013). VCAM1 expression correlated with tumorigenesis and poor prognosis in high grade serous ovarian cancer. Am. J. Transl. Res..

[27] Shah N (2012). Prognostic value of serum CD44, intercellular adhesion molecule-1 and vascular cell adhesion molecule-1 levels in patients with indolent non-Hodgkin lymphomas. Leuk Lymph..

[28] O'Hanlon DM (2002). Soluble adhesion molecules (E-selectin, ICAM-1 and VCAM-1) in breast carcinoma. Eur. J. Cancer.

[29] Silva HC, Garcao F, Coutinho EC, De Oliveira CF, Regateiro FJ (2006). Soluble VCAM-1 and E-selectin in breast cancer: relationship with staging and with the detection of circulating cancer cells. Neoplasma.

[30] Tempia-Caliera AA (2002). Adhesion molecules in human pancreatic cancer. J. Surg. Oncol..

[31] Ye H (2018). Tumor-associated macrophages promote progression and the Warburg effect via CCL18/NF-kB/VCAM-1 pathway in pancreatic ductal adenocarcinoma. Cell Death Dis..

[32] Miyabayashi K (2013). Erlotinib prolongs survival in pancreatic cancer by blocking gemcitabine-induced MAPK signals. Cancer Res..

[33] Ijichi H (2011). Inhibiting Cxcr2 disrupts tumor-stromal interactions and improves survival in a mouse model of pancreatic ductal adenocarcinoma. J. Clin. Invest..

[34] Garton KJ (2003). Stimulated shedding of vascular cell adhesion molecule 1 (VCAM-1) is mediated by tumor necrosis factor-alpha-converting enzyme (ADAM 17). J. Biol. Chem..

[35] Tokuhira M (2000). Soluble vascular cell adhesion molecule 1 mediation of monocyte chemotaxis in rheumatoid arthritis. Arthr. Rheum..

[36] Mitchem JB (2013). Targeting tumor-infiltrating macrophages decreases tumor-initiating cells, relieves immunosuppression, and improves chemotherapeutic responses. Cancer Res..

[37] Tidball JG (2017). Regulation of muscle growth and regeneration by the immune system. Natl. Rev. Immunol..

[38] Clark CE (2007). Dynamics of the immune reaction to pancreatic cancer from inception to invasion. Cancer Res..

[39] Reni M (2009). Carbohydrate antigen 19–9 change during chemotherapy for advanced pancreatic adenocarcinoma. Cancer.

[40] Iademarco MF, McQuillan JJ, Rosen GD, Dean DC (1992). Characterization of the promoter for vascular cell adhesion molecule-1 (VCAM-1). J. Biol. Chem..

[41] Lee CW (2008). Tumor necrosis factor-alpha enhances neutrophil adhesiveness: induction of vascular cell adhesion molecule-1 via activation of Akt and CaM kinase II and modifications of histone acetyltransferase and histone deacetylase 4 in human tracheal smooth muscle cells. Mol. Pharmacol..

[42] Arlt A (2003). Role of NF-kappaB and Akt/PI3K in the resistance of pancreatic carcinoma cell lines against gemcitabine-induced cell death. Oncogene.

[43] Zhang Z (2016). Gemcitabine treatment promotes pancreatic cancer stemness through the Nox/ROS/NF-kappaB/STAT3 signaling cascade. Cancer Lett..

[44] Hess V (2008). CA 19–9 tumour-marker response to chemotherapy in patients with advanced pancreatic cancer enrolled in a randomised controlled trial. Lancet Oncol..

[45] Hammad N (2010). CA19-9 as a predictor of tumor response and survival in patients with advanced pancreatic cancer treated with gemcitabine based chemotherapy. Asia Pac. J. Clin. Oncol..

[46] Nakai Y (2008). CA 19–9 response as an early indicator of the effectiveness of gemcitabine in patients with advanced pancreatic cancer. Oncology.

[47] Tempero MA (1987). Relationship of carbohydrate antigen 19–9 and Lewis antigens in pancreatic cancer. Cancer Res..

[48] Von Hoff DD (2013). Increased survival in pancreatic cancer with nab-paclitaxel plus gemcitabine. N. Engl. J. Med..

[49] Conroy T (2011). FOLFIRINOX versus gemcitabine for metastatic pancreatic cancer. N. Engl. J. Med..

[50] Melisi D (2011). Modulation of pancreatic cancer chemoresistance by inhibition of TAK1. J. Natl. Cancer Inst..

[51] Sano M (2019). Blocking CXCLs-CXCR2 axis in tumor-stromal interactions contributes to survival in a mouse model of pancreatic ductal adenocarcinoma through reduced cell invasion/migration and a shift of immune-inflammatory microenvironment. Oncogenesis.

[52] Yamamoto K (2014). Loss of histone demethylase KDM6B enhances aggressiveness of pancreatic cancer through downregulation of C/EBPalpha. Carcinogenesis.

