# Supplementary material for: Soluble VCAM-1 promotes gemcitabine resistance via macrophage infiltration and predicts therapeutic response in pancreatic cancer
Source: Sci Rep. 2020 Dec 3;10:21194. doi: 10.1038/s41598-020-78320-3 (PMC7713301; doi:10.1038/s41598-020-78320-3)
Supplement: Supplementary file 1 — Supplementary information. [file 41598_2020_78320_MOESM1_ESM.pdf]

Soluble VCAM-1 promotes gemcitabine resistance via macrophage infiltration and predicts therapeutic response in pancreatic cancer

Ryota Takahashi, Hideaki Ijichi, Makoto Sano, Koji Miyabayashi, Dai Mohri, Jinsuk Kim, Gen Kimura, Takuma Nakatsuka, Hiroaki Fujiwara, Keisuke Yamamoto, Yotaro Kudo, Yasuo Tanaka, Keisuke Tateishi, Yousuke Nakai, Yasuyuki Morishita, Katsura Soma, Norihiko Takeda, Harold L Moses, Hiroyuki Isayama, Kazuhiko Koike

Supplementary Figure 1

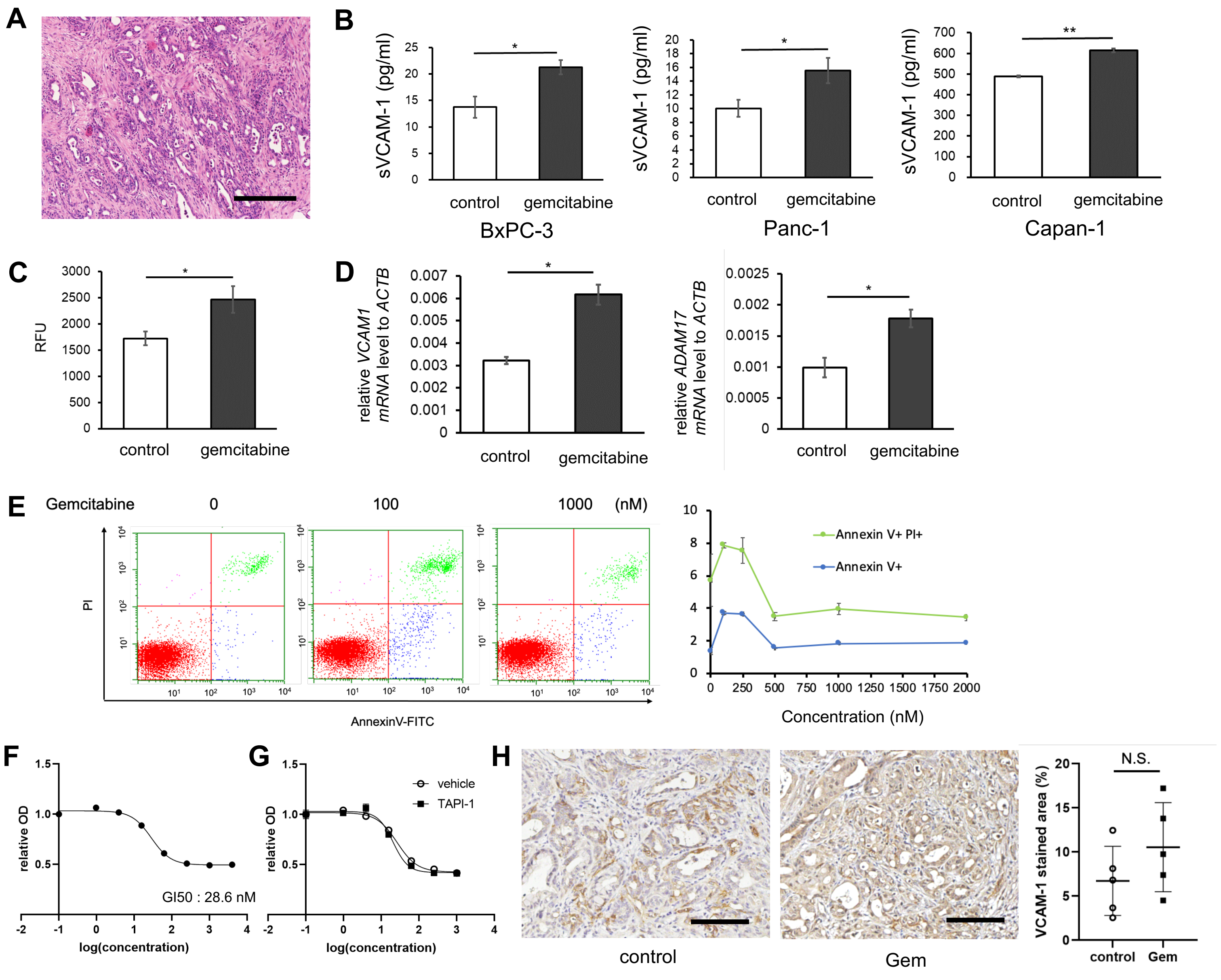

Supplementary Figure1.

(A) Representative image of H&E staining of PDAC from PKF mice at 8 weeks of age. Scale bar, 200 µm. (B) Bar graphs showing sVCAM-1 level in the supernatant of indicated human PDAC cell lines with or without gemcitabine treatment (1 µM) for 24 h, measured by ELISA (n=4 each). Mean±SEM. *: p<0.05, **: p<0.005. (C) Bar graph showing ADAM17 activity in Capan-1 cells cultured with or without gemcitabine (1 µM) for 24 h (n=4 each). The result is shown as relative fluorescence unit (RFU). Mean±SEM. *: p<0.05. (D) Relative quantification of *VCAM1* and *ADAM17* mRNA expression in Capan-1 cells cultured with or without gemcitabine (1 µM) for 24 h (n=4 each). Mean±SEM. *: p<0.05, **: p<0.005. (E) Representative dot plot images from flowcytometry of Annexin V+ and PI+ cells within K399 cells treated with gemcitabine for 24 hours. Right panel is showing quantification of each population at indicated concentrations of gemcitabine. Mean±SD. (F) Dose-response curve showing relative viability of K399 cells treated with gemcitabine at indicated concentrations for 24 hours. Mean±SD. (G) Dose-response curve showing relative viability of K399 cells treated with gemcitabine at indicated concentrations for 24 hours, following pretreatment with 50 µM TAPI-1 or vehicle for 1 hour. Mean±SD. (H) Representative images of IHC for VCAM-1 in PDAC from PKF mice untreated or treated with gemcitabine for 3 weeks starting from 4 weeks of age (n=5). Mean±SD. N.S., not significant.

Supplementary Figure 2

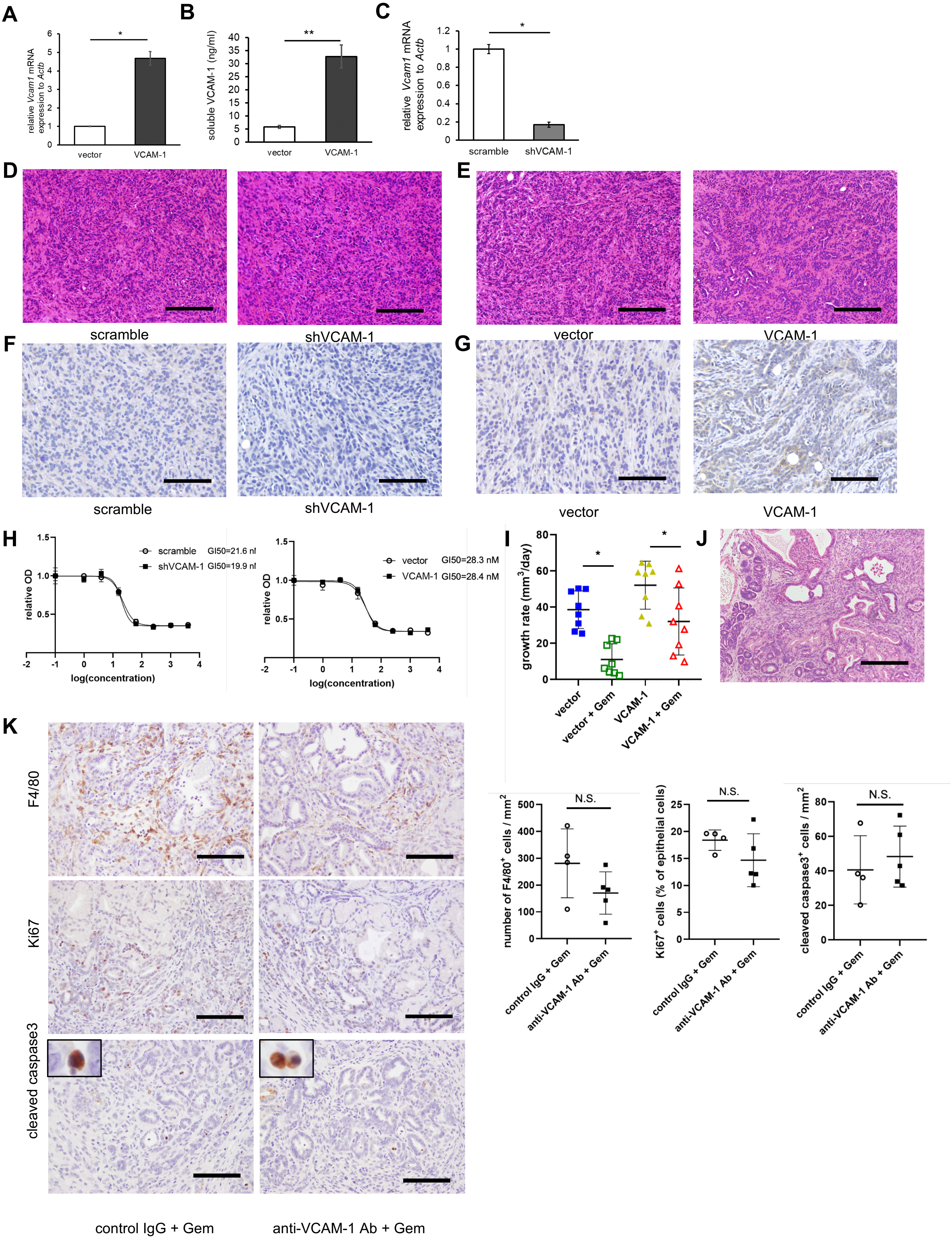

Supplementary Figure 2.

(A) Relative quantification of *Vcam1* mRNA expression in K399 cells overexpressing VCAM-1 or infected with control vector (n=4 each). Mean±SEM. *: $p<0.05$. (B) Bar graph showing soluble VCAM-1 level in the supernatant of K399 cells overexpressing VCAM-1 or infected with control vector, measured by ELISA (n=4 each). Mean±SEM. **: $p<0.005$. (C) Relative quantification of *Vcam1* mRNA expression in K399 cells infected with shVCAM-1 or scramble shRNA (n=4 each). Mean±SEM. *: $p<0.05$. N.S.: not significant. (D) Representative pictures of H&E staining in allograft tumors by K399 cells infected with shVCAM-1 or scramble shRNA. Scale bars, 100 µm. (E) Representative pictures of H&E staining in allograft tumors by K399 cells overexpressing VCAM-1 or infected with control vector. Scale bars, 100 µm. (F) Representative pictures of IHC for VCAM-1 in allograft tumors by K399 cells infected with shVCAM-1 or scramble shRNA. Scale bars, 50 µm. (G) Representative pictures of IHC for VCAM-1 in allograft tumors by K399 cells overexpressing VCAM-1 or infected with control vector. Scale bars, 50 µm. (H) Dose-response curve showing relative viability of K399 cells infected with shVCAM-1 or scramble shRNA (left) and K399 cells overexpressing VCAM-1 or infected with control vector (right) treated with gemcitabine at indicated concentrations for 24 hours. Mean±SD. (I) Dot plot of tumor growth rate from day 11 to day 15 calculated from the data in Fig. 2C. Mean±SD. *: $p<0.05$. (J) Representative picture of H&E staining of PanIN2-3 lesions observed in PKF mice at 4 weeks old. Scale bar, 100 µm. (K) Representative pictures of IHC for F4/80, Ki67, and cleaved caspase3 in PDAC from PKF mice treated as shown Fig. 2G. Insets are showing examples of cleaved caspase3^{+} cells. Dot plots are showing quantitative analysis of the staining. Scale bars, 100 µm. Mean±SD. N.S., not significant.

Supplementary Figure 3

A

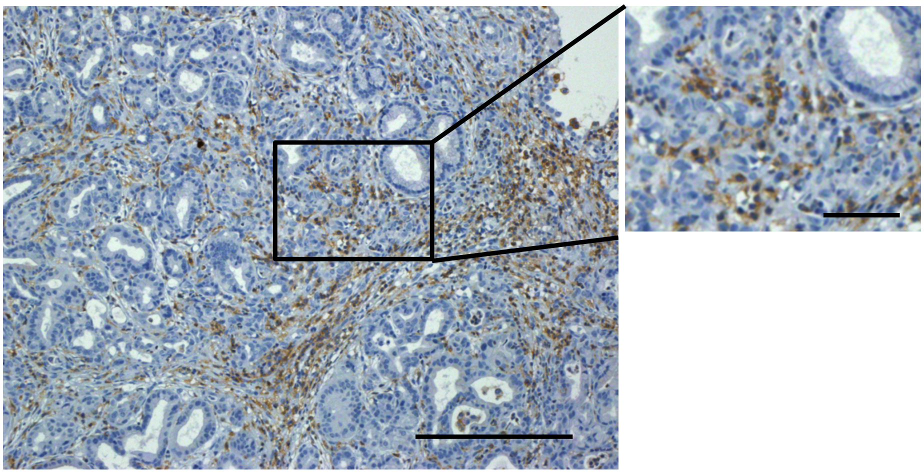

B

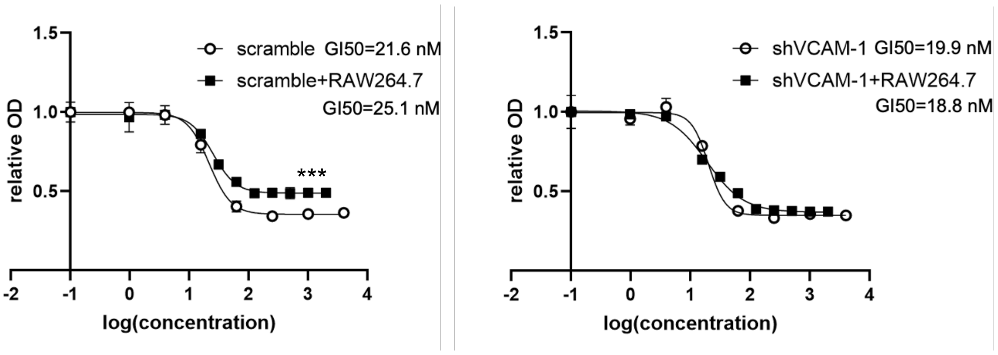

Supplementary Figure 3.

(A) Representative image of IHC for F4/80 in PDAC of PKF mice. Magnified image of indicated area in the left panel is shown in the right. Scale bar: 200 µm (left), 50 µm (right). (B) Dose-response curve showing relative viability of K399 cells infected with shVCAM-1 (right) or scramble shRNA (left) which are cocultured with RAW264.7 cells and treated with gemcitabine at indicated concentrations for 24 hours. Mean±SD. ***: $p<0.001$.

Supplementary Figure 4

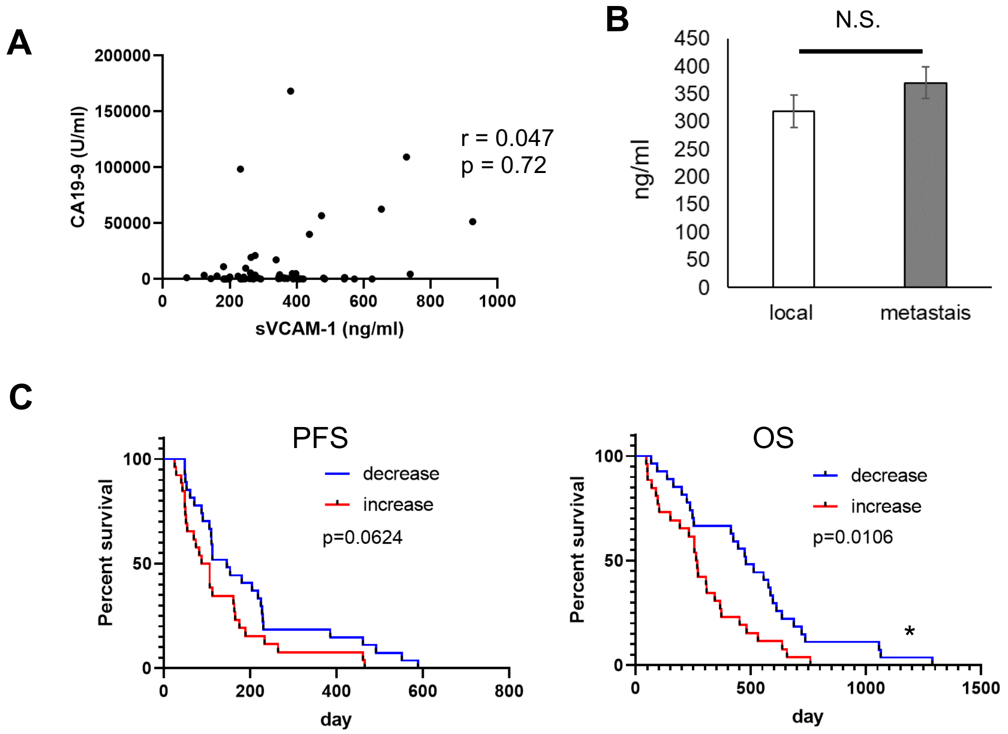

Supplementary Figure 4.

(A) Scatter plot graph showing correlation of CA19-9 levels and sVCAM-1 levels in the plasma of patients at the beginning of gemcitabine treatment (n=57). (B) Bar graph showing soluble VCAM-1 levels in the plasma of patients with locally advanced disease (n=17) or metastatic disease (n=40). Mean±SEM. N.S., not significant. (C) Kaplan-Meier curve showing correlation of change in CA19-9 in the first 4 weeks of chemotherapy with PFS (left) and OS (right). *: $p<0.05$.

Supprementary Table 1. List of cytokines/chemokines shown in cytokine array

1<br>2	POS	POS	NEG	NEG	BLANK	Axl	BLC (CXCL13)	CD30 Ligand	CD30	CD40	CRG-2	CTACK (CCL27)	CXCL16	Eotaxin-1 (CCL11)
3<br>4	Eotaxin-2 (CCL24)	Fas Ligand	Fractalkine	GCSF	GM-CSF	IFN-gamma	IGFBP-3	IGFBP-5	IGFBP-6	IL-1 alpha	IL-1 beta	IL-2	IL-3	IL-3 R beta
5<br>6	IL-4	IL-5	IL-6	IL-9	IL-10	IL-12 p40/p70	IL-12 p70	IL-13	IL-17A	KC (CXCL1)	Leptin R	Leptin	LIX	L-Selectin
7<br>8	Lymphotactin	MCP-1 (CCL2)	MCP-5	M-CSF	MIG (CXCL9)	MIP-1 alpha	MIP-1 gamma	MIP-2	MIP-3 beta	MIP-3 alpha	PF-4 (CXCL4)	P-Selectin	RANTES	SCF
9<br>10	SDF-1 alpha	TARC (CCL17)	I-309 (CCL1)	TECK (CCL25)	TIMP-1	TNF alpha	TNF RI	TNF RII	TPO	VCAM-1	VEGF-A	BLANK	BLANK	POS

Supprementary Table 2. Quantitative data of the cytokine array

	WT			PKF		
	0 h	4 h	48 h	0 h	4 h	48 h
Axl	0.0088	0.0253	0.0036	0.0189	0.0224	0.0224
BLC	0.0099	0.0148	0.0042	0.0413	0.0135	0.0246
CD30 Ligand	0.0018	0.0069	N.D.	0.0009	N.D.	0.0036
CD30	0.0052	0.0118	N.D.	0.0035	0.0144	0.0053
CD40	0.0003	0.0032	N.D.	N.D.	N.D.	N.D.
CRG-2	0.0011	0.0034	N.D.	N.D.	N.D.	N.D.
CTACK	0.0105	0.0123	0.0042	0.0042	0.0098	0.0128
CXCL16	0.0245	0.0325	0.0158	0.0815	0.1072	0.1182
Eotaxin-1	0.0146	0.0194	0.0069	N.D.	0.0072	0.0063
Eotaxin-2	0.2555	0.2490	0.2038	0.2995	0.2791	0.3422
Fas Ligand	0.0300	0.0282	0.0167	0.0455	0.0549	0.0573
Fractalkine	0.0074	0.0080	N.D.	0.0122	0.0134	0.0193
GCSF	0.0037	0.0071	N.D.	0.0198	0.0142	0.0135
GM-CSF	0.0053	0.0085	N.D.	0.0125	0.0078	0.0203
IFNγ	0.0023	0.0055	N.D.	0.0070	0.0064	0.0129
IGFBP-3	0.0432	0.0888	0.0495	0.1717	0.1346	0.1708
IGFBP-5	0.0185	0.0249	0.0149	0.0278	0.0258	0.0502
IGFBP-6	0.1094	0.1733	0.1141	0.2467	0.2622	0.2505
IL-1α	0.0190	0.0270	0.0179	0.0295	0.0399	0.0501
IL-1β	0.0016	0.0018	N.D.	N.D.	0.0065	0.0025
IL-2	0.0089	0.0087	0.0035	0.0038	0.0128	0.0138
IL-3	0.0003	N.D.	N.D.	N.D.	0.0157	0.0038
IL-3Rβ	0.0057	0.0056	N.D.	0.0007	0.0318	0.0204
IL-4	0.0147	0.0168	0.0079	0.0217	0.0303	0.0432
IL-5	0.0053	0.0049	N.D.	0.0082	0.0223	0.0146
IL-6	N.D.	N.D.	N.D.	0.0106	0.0112	0.0038
IL-9	0.0036	0.0077	N.D.	0.0101	0.0131	0.0181
IL-10	N.D.	0.0030	N.D.	0.0064	0.0116	0.0113
IL-12 p40/p70	0.0171	0.0496	0.0124	0.0540	0.0587	0.0946
IL-12 p70	0.0089	0.0166	0.0042	0.0196	0.0219	0.0444
IL-13	0.0011	0.0041	N.D.	0.0026	0.0037	0.0187
IL-17A	N.D.	N.D.	N.D.	N.D.	0.0064	0.0054
KC	0.0002	0.0040	N.D.	0.0007	0.0065	0.0159
Leptin R	0.0131	0.0215	0.0120	0.0168	0.0353	0.0355
Leptin	0.0116	0.0121	0.0058	0.0041	0.0295	0.0207
LIX	0.1231	0.1232	0.0896	0.1225	0.2158	0.1451
L-Selectin	0.5362	0.5697	0.5117	1.2682	2.7381	2.6507
Lymphotactin	0.0666	0.0898	0.0632	0.1087	0.1074	0.1637
MCP-1	0.0094	0.0139	0.0062	0.0180	0.0254	0.0465
MCP-5	0.0020	0.0075	N.D.	0.0053	0.0067	0.0216
M-CSF	0.0111	0.0228	0.0116	0.0282	0.0341	0.0574
MIG	0.0337	0.0493	0.0403	0.0468	0.0565	0.0546
MIP-1α	0.0042	0.0094	0.0014	0.0094	0.0186	0.0395
MIP-1γ	0.3176	0.4295	0.3409	0.4927	0.6444	0.8224
MIP-2	0.0232	0.0294	0.0181	0.0363	0.0423	0.0776
MIP-3β	0.0487	0.0594	0.0591	0.0517	0.0748	0.0903
MIP-3α	0.0125	0.0200	0.0128	0.0117	0.0299	0.0326
PF-4	0.1760	0.1809	0.1118	0.2113	0.1612	0.1692
P-Selectin	0.1144	0.1930	0.1345	0.1984	0.3369	0.3177
RANTES	0.0107	0.0143	0.0061	0.0039	0.0474	0.0226
SCF	0.0132	0.0143	0.0073	0.0010	0.0592	0.0200
SDF-1α	0.0437	0.0627	0.0292	0.0856	0.1207	0.1580
TARC	0.0066	0.0077	N.D.	0.0102	0.0183	0.0368
I-309	0.0112	0.0189	0.0048	0.0219	0.0317	0.0575
TECK	N.D.	0.0005	N.D.	N.D.	N.D.	0.0068
TIMP-1	N.D.	0.0054	N.D.	0.0351	0.0403	0.0421
TNFα	0.0059	0.0097	0.0003	0.0091	0.0251	0.0310
TNF RI	0.1226	0.1758	0.1029	0.3747	0.3712	0.6429
TNF RII	0.0371	0.0800	0.0270	0.1635	0.2005	0.2521
TPO	0.0370	0.0470	0.0375	0.0403	0.0731	0.0910
VCAM-1	0.5298	0.5258	0.5424	0.5973	0.8837	0.9806
VEGF-A	0.0202	0.0341	0.0250	0.0171	0.0379	0.0452

N.D., not detected

Supprementary Table 3. Primer sequences used in qRT-PCR

Vcam1	Forward	5'-TGACAAGTCCCCATCGTTGA-3'
	Reverse	5'-ACCTCGCGACGGCATAATT-3'
Adam17	Forward	5'-CCCCCACCCGGAGATGCTGA-3'
	Reverse	5'-CGGCACACACGGGCCAGAAA-3'
Actb	Forward	5'-TGACAGGATGCAGAAGGAGA-3'
	Reverse	5'-GCTGGAAGGTGGACAGTGAG-3'
VCAM1	Forward	5'-CAAAGGCAGAGTACGCAAACAC-3'
	Reverse	5'-GCTGACCAAGACGGTTGTATCTC-3'
ADAM17	Forward	5'-CAGCTGGAGTCCTGTGCATGT-3'
	Reverse	5'-ACACAGCGGCCAGAAAGGT-3'
ACTB	Forward	5'-AAGGAGCCCCACGAGAAAAAT-3'
	Reverse	5'-ACCGAACTTGCATTGATTCCAG-3'
